# Dysplastic Hepatocytes Develop Nuclear Inclusions in a Mouse Model of Viral Hepatitis

**DOI:** 10.1371/journal.pone.0099872

**Published:** 2014-06-16

**Authors:** Priyanka Thakur, Folami Lamoke, Joanna M. Chaffin, Manuela Bartoli, Jeffrey R. Lee, Michael B. Duncan

**Affiliations:** 1 Section of Gastroenterology/Hepatology, Department of Medicine, Medical College of Georgia, Georgia Regents University, Augusta, Georgia, United States of America; 2 Department of Pharmacology, Medical College of Georgia, Georgia Regents University, Augusta, Georgia, United States of America; 3 Department of Ophthalmology, Medical College of Georgia, Georgia Regents University, Augusta, Georgia, United States of America; 4 Department of Pathology, Medical College of Georgia, Georgia Regents University, Augusta, Georgia, United States of America; 5 Georgia Regents University Cancer Center, Medical College of Georgia, Georgia Regents University, Augusta, Georgia, United States of America; 6 Department of Biochemistry and Molecular Biology, Medical College of Georgia, Georgia Regents University, Augusta, Georgia, United States of America; 7 Charlie Norwood Veterans Affairs Medical Center, Augusta, Georgia, United States of America; Drexel University College of Medicine, United States of America

## Abstract

Viral hepatitis resulting in chronic liver disease is an important clinical challenge and insight into the cellular processes that drive pathogenesis will be critical in order to develop new diagnostic and therapeutic options. Nuclear inclusions in viral and non-viral hepatitis are well documented and have diagnostic significance in some disease contexts. However, the origins and functional consequences of these nuclear inclusions remain elusive. To date the clinical observation of nuclear inclusions in viral and non-viral hepatitis has not been explored at depth in murine models of liver disease. Herein, we report that in a transgenic model of hepatitis B surface antigen mediated hepatitis, murine hepatocytes exhibit nuclear inclusions. Cells bearing nuclear inclusions were more likely to express markers of cell proliferation. We also established a correlation between these inclusions and oxidative stress. N-acetyl cysteine treatment effectively reduced oxidative stress levels, relieved endoplasmic reticulum (ER) stress, and the number of nuclear inclusions we observed in the transgenic mice. Our results suggest that the presence of nuclear inclusions in hepatocytes correlates with oxidative stress and cellular proliferation in a model of antigen mediated hepatitis.

## Introduction

Nuclear inclusions are a relatively rare histological occurrence and are broadly defined as the accumulation of substances in the nuclear matrix, which are not normally of nuclear origin. These inclusions are associated with various clinical conditions including cancer, obesity, diabetes, nonalcoholic fatty liver disease, Wilson disease, and amiodarone toxicity and observed in many tissue types including liver [1], [2], [3], [4]. The origins of these inclusions are unknown, however their presence has been used in histo-pathological diagnosis. For example, pseudo-inclusions among thyroid tumors are characteristic microscopic lesions of papillary thyroid carcinoma and their presence is considered in diagnosis of this tumor [5], [6]. Accumulations of homogeneous eosinophilic material in these inclusions, are often enriched in glycogen and are sometimes referred to as glycogenated nuclei [3].

While having some histo-pathological value, the functional role of nuclear inclusions in disease progression remains unclear. A recent report suggests that these inclusions correlate strongly with cellular senescence, and DNA damage markers in various forms of liver disease including chronic viral hepatitis [7]. Viral hepatitis, including hepatitis B, is an important promoter of chronic liver disease and a common initiating event of hepatocellular carcinoma in the developing world. While the pathogenesis of viral hepatitis is becoming clear, further studies are required to gain a complete understanding of how the disease progresses [8]. Several murine models of viral antigen induced hepatitis have been developed and used to study disease mechanisms [9], [10]. We wanted to determine if a transgenic mouse overexpressing the hepatitis B surface antigen develop nuclear inclusions as has been reported in human liver tissue [11]. Herein, we present data indicating that dysplastic hepatocytes arising from hepatitis B surface antigen-mediated injury are prone to develop nuclear inclusions and this process may be dependent on oxidative stress associated with the chronic injury response.

## Materials and Methods

All reagents were purchased from Fisher Scientific unless otherwise noted.

### Animal care and use

All mice were housed under standard conditions in the Georgia Regents University Animal Research Facility. All experiments were conducted with the ethical approval of the Institutional Animal Care and Use Committee of GRU. C57BL/6J-Tg(Alb1HBV)44Bri/J transgenic mice (HBsAg-Tg) were a kind gift from Dr. Yukai He (GRU) (MGI Ref ID J:86165). C57BL/6J-Tg (Alb1HBV)44Bri/J transgenic mice over-express the hepatitis B virus large envelope polypeptide, accumulating large quantities of hepatitis B surface antigen (HBsAg) within the hepatocyte [12]. Mice were genotyped as described by The Jackson Laboratory (Stock number: 002226) and littermates not expressing the transgene were used as control.

### Liver Histology and Immunohistochemical analysis

Transgenic and control livers were harvested from mice at specific time points and were either fixed in 4% paraformaldehyde (PFA) or snap frozen in optimal cutting temperature (OCT) compound using liquid nitrogen. PFA fixed tissue was embedded in paraffin and sections were prepared and mounted on slides by Pathology Research Services (GRU). For morphometric studies, sections were stained with haemotoxylin and eosin (H&E) or periodic acid Schiff's (PAS, Sigma) staining. The number of PAS positive inclusions and total inclusions were counted manually in five transgenic mice/HPF. To determine glycogen content, an enzymatic assay was done to digest glycogen, using alpha-amylase (Sigma-Aldrich) by Pathology Research Services (GRU). Photographs for H&E and PAS staining were taken with a Zeiss Axioplan 2 microscope.

The average nuclear area was calculated in 6 wild type mice (2 wild type mice from each time point, 3month. 6 month, 9 month), three 3-month and 6-month transgenic mice and four 9-month transgenic mice (10 high powered fields/mouse) using image J. Number of nuclear inclusions were calculated in transgenic mice at 3 and 6 and 9-month interval (n = 3, 5 high powered fields/mouse). All the large hepatocytes and ground glass hepatocytes with or without nuclear inclusions were manually counted in 9 month transgenic (n = 5, all hepatocytes/high powered field).

For 4-Hydroxy-nonenal (4-HNE, polyclonal goat, Abcam) immuno-fluorescence staining, OCT embedded liver tissue was sectioned by cryostat at 5-micron thickness and air-dried. Sections were fixed in ice-cold acetone for 20 minutes, blocked with 1% bovine serum albumin in tris-buffered saline, and then incubated with 4-HNE antibody (1∶200) overnight at 4°C in a humid chamber. Sections were washed with Tris-buffered saline and incubated with Alexa Fluor 546 conjugated anti-goat secondary antibody (1∶500 dilution) for one hour at room temperature, counterstained with DAPI, and mounted with MOWIOL mounting media. Images were captured on the Zeiss Axioplan 2 epifluorescence microscope. Five transgenic mice and five NAC treated mice were used to analyze relative fluorescence intensity (5 high powered fields/mouse) for 4-HNE immuno-histochemistry. Also, Total nuclear inclusions were counted (5 high powered fields/nuclei) in transgenic mice (n = 5) and NAC treated transgenic mice (n = 5).

To examine hepatitis B virus large envelope polypeptide expression in transgenic mice, paraffin sections from five transgenic mice were stained with hepatitis B surface Ag antibody (clone 3E7, Santa Cruz Biotechnology). This antibody recognizes the ‘a’ determinant known to be present on all Hepatitis B surface antigen. HBsAg staining was analysed in 5 high-powered fields/mouse. All hepatocytes with or without inclusions, positive or negative for HBsAg expression were counted.

Paraffin sections were used to detect proliferating cell nuclear antigen by immuno-fluorescence. Sections were stained for PCNA (mouse, Millipore) by using Mouse on mouse (M.O.M.) blocking kit Vector lab. Secondary staining was done with biotin-conjugated anti-mouse IgG and tertiary staining with Alexa flour 555 (Invitrogen) conjugated streptavidin. For immuno-histochemical quantifications, 5 images per slide were captured across the whole section at 20× magnification. The areas with vacuolated and non-vacuolated nuclei, positive for PCNA, were identified manually and quantified by ImageJ.

Similarly, horseradish peroxidase based immuno-histological staining on paraffin sections for calnexin (goat, Santa Cruz, Dallas, TX) and histone H3 (rabbit, Cell Signaling, Danvers, MA), was done and visualized by 3,3′-diaminobenzidine (DAB) staining. Slides were counterstained with hematoxylin. Histone H3 and calnexin positive inclusions verses total inclusions were counted in five transgenic mice (all inclusions/high powered field)

Histopathological assessments were carried out by two independent pathologists evaluating the relative percentage of enlarged nuclei (large cell change/dysplasia), small cell dysplasia, ground glass change, steatosis, necrosis, and inflammation.

### NAC (N- acetyl cysteine) treatment

Transgenic mice were randomly assigned to ad lib drinking water supplemented with 40 millimolar NAC (N-acetyl cysteine) at the age of 9–10 month and remained on supplemented water until sacrifice. At the end of four weeks mice were sacrificed and tissue were harvested for analysis.

### Western Blotting

Protein lysates for western blot, were isolated from transgenic and NAC treated mice livers and aliquots were separated on the SDS-PAGE. Electrophoresed proteins were transferred to polyvinylidene fluoride (PVDF) membrane and the membranes were blocked in 5% milk in Tris-buffered saline with 0.1% tween 20 (TBST). Blots were incubated overnight at 4°C with primary antibody, Chop (mouse, Santa Cruz, 1∶400) in 5%BSA/TBST. Then membranes were washed and incubated with appropriate HRP- conjugated secondary antibodies. Protein bands were visualized using ECL chemiluminescence (Bio-Rad). Equal loading was confirmed by stripping the membrane (Tris/SDS/beta mercaptoethanol) and re-probing for beta-actin (1∶1000, Thermo).

### TUNEL Assay

Terminal deoxynucleotidyl transferase-mediated nick end-labeling (TUNEL) assay was performed using kits purchased from Promega according to manufacturer's protocols. Paraffin sections of livers of 9–10 month old mice were used for TUNEL Assay.

### Senescence associated beta-gal assay

Fresh frozen tissue sections (8–10 micron thick) were used for senescence-associated beta galactosidase staining according to the manufacturer's protocols (Cell Signaling Technology).

### Statistical analysis

Data are expressed as the mean ±standard error of the mean. Statistical significance was determined by a paired sample Student's *t* test using a one- or two-tailed distribution. Values of p<0.05 were considered significant.

## Results

### Nuclear inclusions in HBsAg-Tg mice

**Figure 1:**
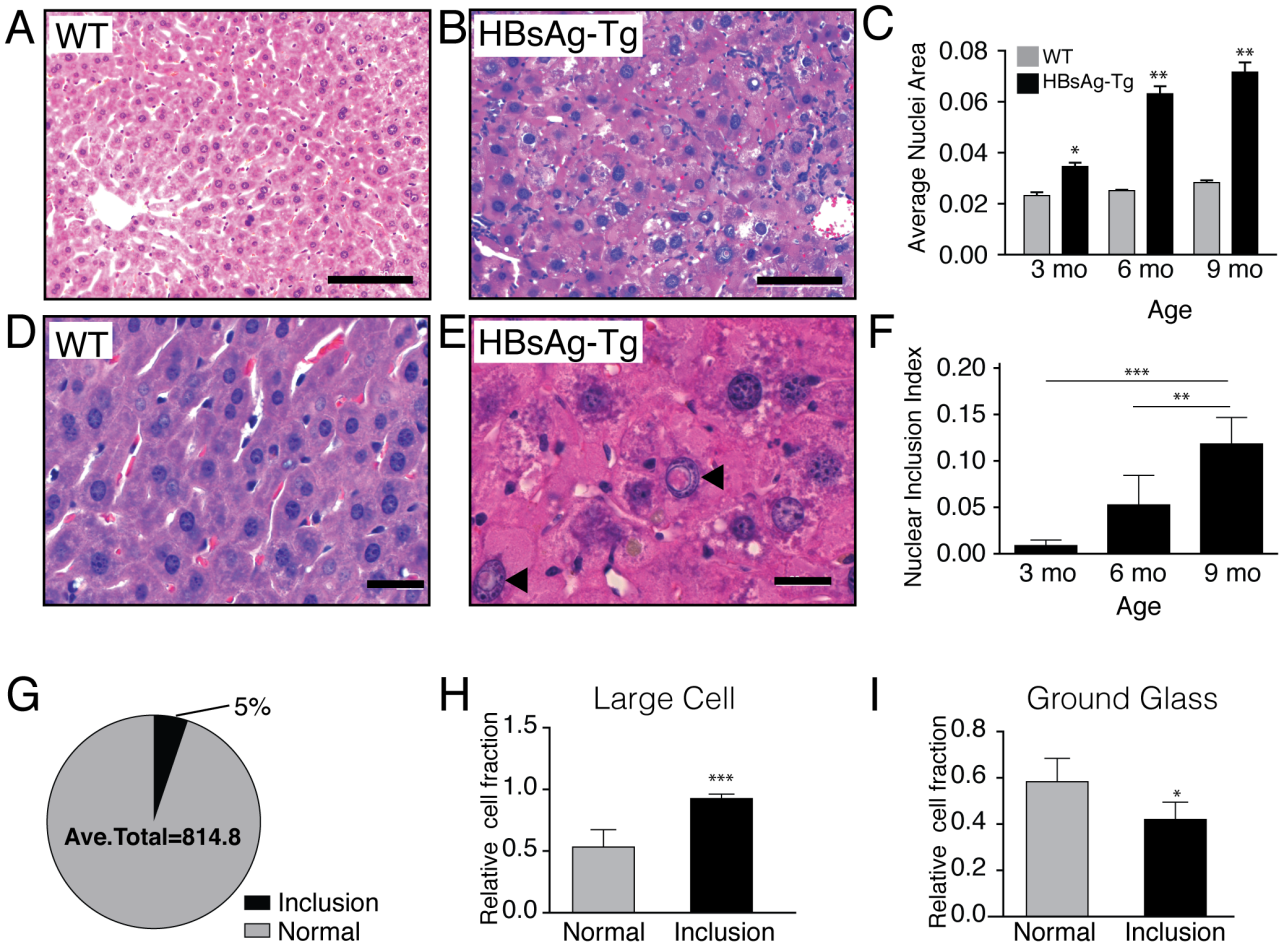
Nucleus size and inclusions in wild type (WT) and nine month old HBsAg-Tg mice. (**A**) Hematoxylin and eosin staining of wild type liver, showing hepatocyte with normal nuclei. In contrast, (**B**) eosinophilic rich nuclear inclusions with larger nuclei can be seen in liver tissue of HBsAg-Tg mice. (**C**) The size of hepatocyte nuclei increases at 3 months (n = 3), 6 months (n = 3), and 9 months (n = 4) of age compared to wild type controls (n = 2) at each time point. Higher magnification in (**D**) WT and (**E**) HBsAg-Tg demonstrates inclusions with eosinophilic material. (**F**) The ratio of cells with nuclear inclusions compared to normal nuclei increases at 3 months (n = 3), 6 months (n = 3), and 9 months (n = 5) of age. (**G**) The fraction of hepatocytes with nuclear inclusions was 5% of the total hepatocyte population in HBsAg-Tg mice (n = 5). (**H**) Large cell dysplasia correlated strongly with hepatocytes having nuclear inclusions (n = 5). (**I**) Correlation of ground glass hepatocytes with and without nuclear inclusions (n = 5). *,p<0.05; **,p<0.01; ***,p<0.001; ****, p<0.0001, **A**, **B** scale bar 100 µm; **D**, **E** scale bar 20 µm.

Transgenic mice overexpressing HBsAg serve as a well-established model of antigen-mediated hepatitis [9], [10], [12]. These mice develop chronic injury typified by a persistent inflammatory response, tissue remodeling and regeneration, and eventually neoplastic lesions [13], [14], [15]. In this model, we observed hepatocytes with well-defined nuclear inclusions while wild type littermates did not develop these structures (**Fig. 1A–B**). Swelling hepatocytes and significant enlargement of nuclei were also observed as a primary histological finding (**Fig. 1C**). The nuclear inclusions often contained eosinophilic material of HBsAg-Tg mouse liver tissue that was not found in WT littermates (**Fig. 1D–E**), as early as 3 month of age. The frequency of these nuclear inclusions increased with age (**Fig. 1F**).

### HBsAg expression in transgenic mouse liver

**Figure 2:**
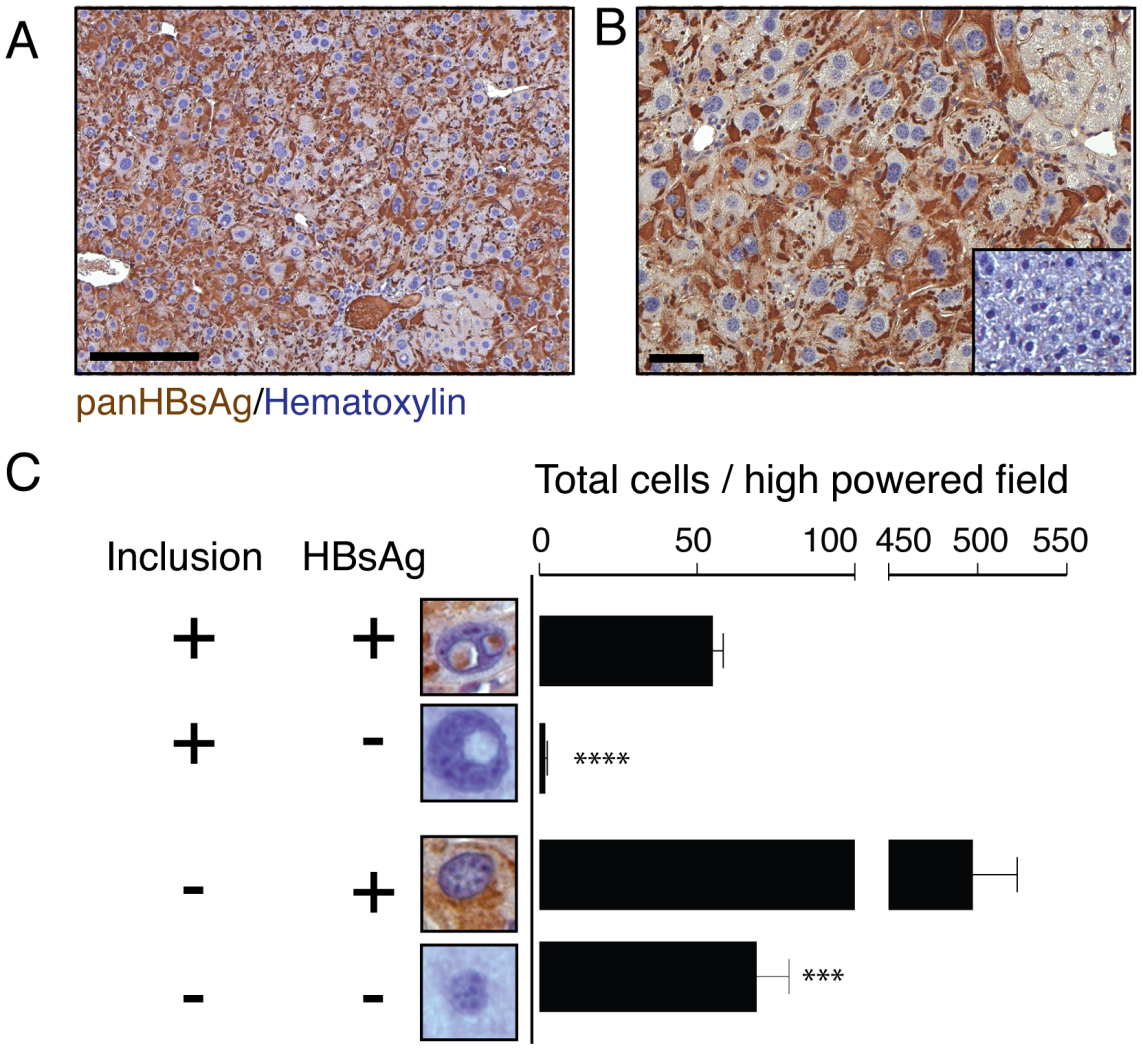
Transgenic hepatitis B virus large envelope polypeptide (HBsAg) expression in hepatocytes with nuclear inclusions. (A, B) A high level of HBsAg expression in the cytoplasm of hepatocytes in nine-month-old transgenic mice. Antibody specificity was confirmed by lack of immuno-reactivity in wild-type control mice shown in the inset of (**B**). (**C**) Quantification of the relative number of hepatocytes from HBsAg transgenic mice that express the HBsAg in inclusion-bearing and normal hepatocytes (n = 5). p<0.01; ***,p<0.001; ****, p<0.0001, scale bar 50 µm.

HBsAg expression in hepatocytes has pleiotrophic effects including alterations in cellular metabolism, oxidative stress, and gene expression [16], [17], [18], [19], [20]. We examined the distribution of hepatitis B virus large envelope polypeptide in transgenic mice and observed expression of the antigen in the cytoplasm of hepatocytes and in liver sinusoids (**Fig. 2A–B**). In addition, we also observed a strong correlation between HBsAg expression in hepatocytes and the presence of nuclear inclusions (**Fig. 2C**). These results suggest that inclusion formation may be linked with hepatitis B virus large envelope polypeptide overexpression.

### Vacuolated nuclei contain glycogen deposits

**Figure 3:**
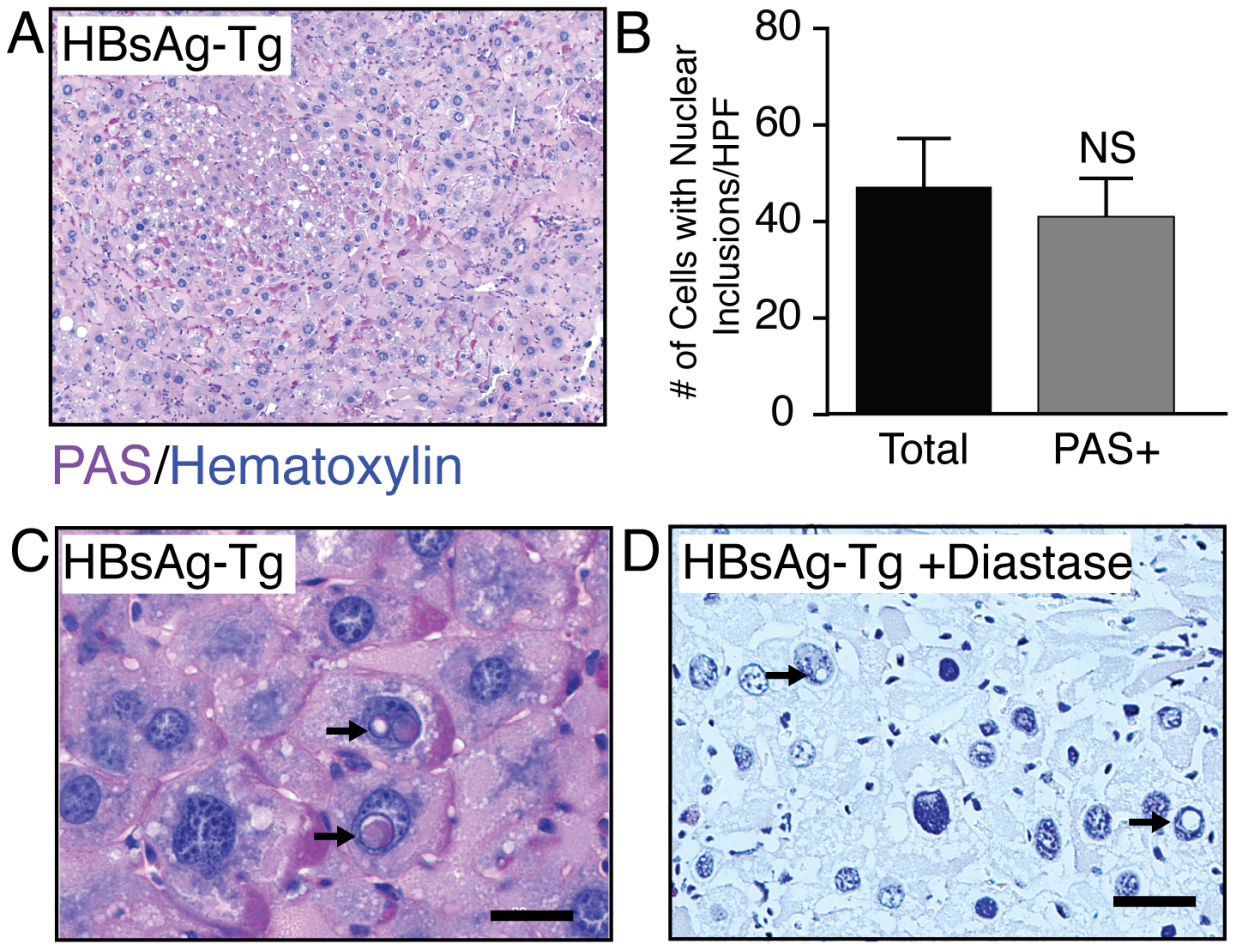
Nuclear inclusions are enriched in glycogen. (**A**) HBsAg-Tg liver nuclear inclusions were enriched for Periodic acid-Schiff (PAS) staining. (B) Hepatocytes bearing nuclear inclusions were typically PAS positive (n = 5). (**C, D**) The material was confirmed to be glycogen by amylase digestion assay. scale bar 20 µm.

Previous reports indicate that nuclear inclusions are typically enriched in glycogen [7], [21]. We wanted to determine if the inclusions we observed in HBsAg-Tg mice contained these glycogen deposits. We performed periodic acid schiff staining (PAS) staining, and observed distinct PAS positive material suggesting that the inclusions are rich in polysaccharides (**Fig. 3A**). Quantification revealed that nearly all hepatocytes bearing nuclear inclusions were PAS positive (**Fig 3B**). Amylase digestion assay confirmed that the primary PAS positive material is glycogen (**Fig. 3C–D**). Non-PAS positive material could also be observed in the inclusions, but its precise nature remains unclear.

### Nuclear inclusions contain cytoplasmic material

**Figure 4:**
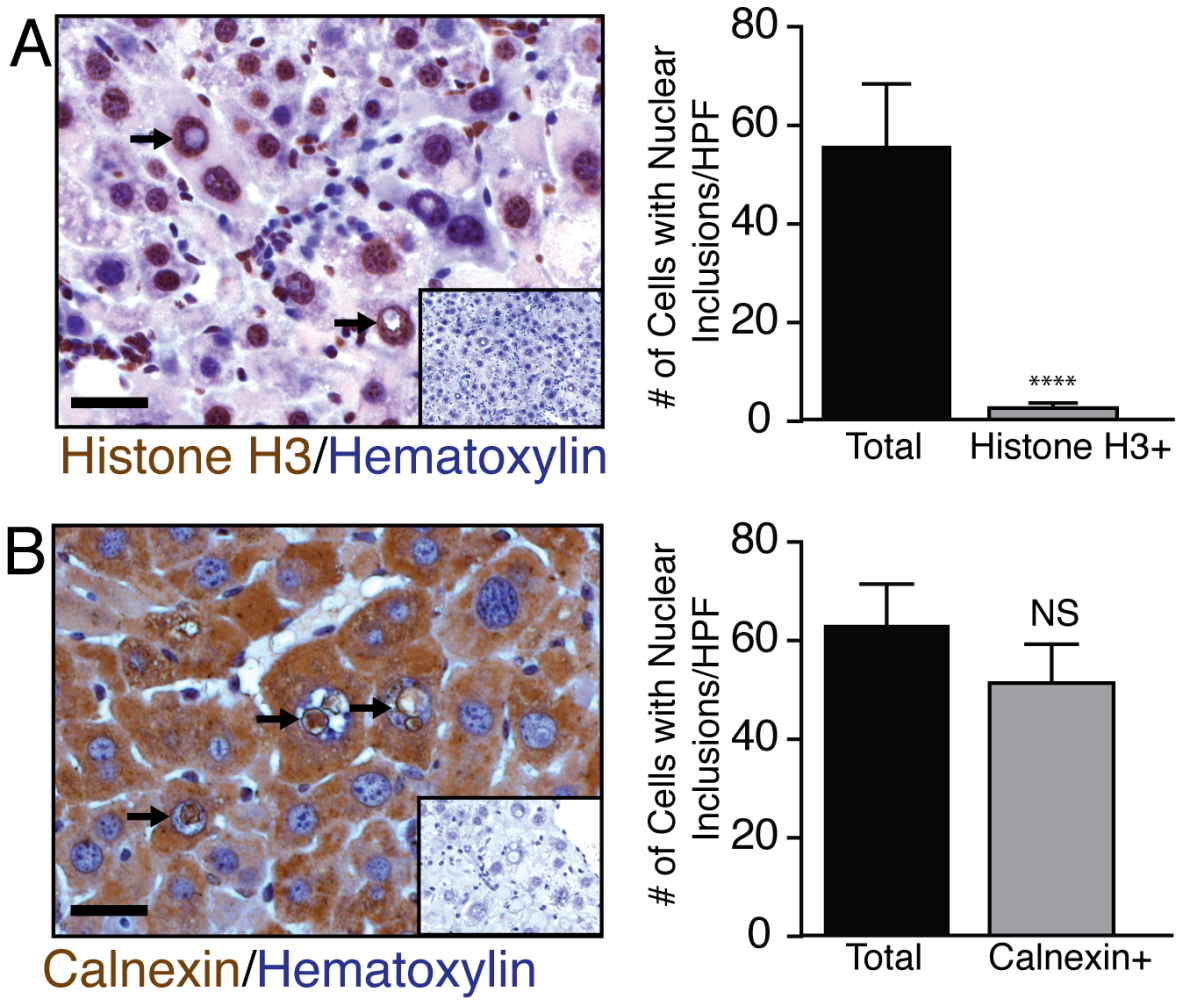
Nuclear inclusions are enriched with endoplasmic reticulum material. The origin of material within these inclusions was examined by Histone H3 and Calnexin staining. (**A**) None of the nuclear inclusions expressed histone H3 (n = 5). (**B**) However, almost all inclusions were clearly positive for calnexin staining (n = 5). Negative controls for Histone H3 and calnexin (no primary antibody) are shown in the insets. scale bar 20 µm.

We wanted to assess the content of the eosinophilic material within the nuclear inclusions. To determine if the inclusions contained nuclear or cytoplasmic material we performed immuno-histochemical staining of two generic makers for the nucleus and cytoplasm. Histone H3 is an important chromatin protein and commonly expressed in most tissue including the liver [22]. In **Fig. 4A**, we observed strong Histone H3 immuno-reactivity in nearly all hepatocyte nuclei with or without nuclear inclusions. We did not observe Histone H3 staining within the inclusions. Calnexin is a common endoplasmic reticulum protein expressed in hepatocytes and may play an important role in hepatitis B envelope protein folding [23], [24]. We observed strong calnexin staining in multiple cell types throughout the HBsAg-Tg mouse liver and the nuclear inclusions were also positive for calnexin (**Fig. 4B**). Calnexin nuclear immuno-reactivity was exclusively localized in the inclusions and did not appear to diffuse into the hematoxylin positive area. From this analysis, we conclude that the nuclear inclusions are predominately composed of cytoplasmic material including endoplasmic reticulum.

### Hepatocytes containing nuclear inclusions express the proliferation marker PCNA

**Figure 5:**
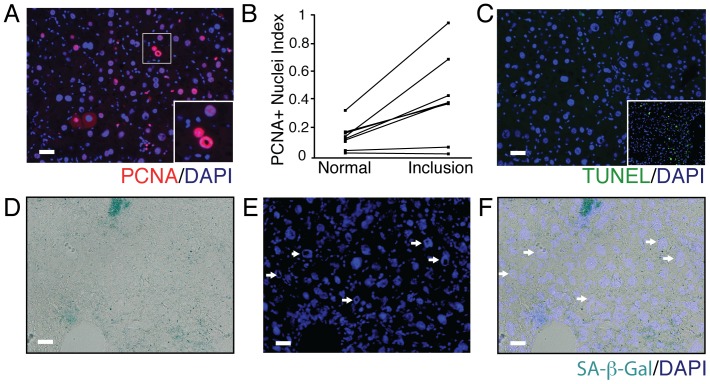
Nuclear inclusions are associated with the proliferative response. (**A**) PCNA positive vacuolated nuclei along with positive PCNA “normal” nuclei could be observed in HBsAg-Tg mice. The inset shows a magnified image of PCNA expressing cells with and with out nuclear inclusions. (**B**) The fraction of PCNA positive with nuclear inclusions was increased in the subset of hepatocytes bearing nuclear inclusions in nine-month-old HBsAg-Tg mice. (**C**) Cell death as determined by TUNEL assay was not a feature of hepatocytes with nuclear inclusions. The inset shows the carbon tetrachloride induced liver injury model (48 hour post intoxication time point) as a positive TUNEL control. (**D-F**) Senescence was rarely observed in the HBsAg-Tg at 9 months and did not correlate with hepatocytes containing nuclear inclusions. scale bar 20 µm.

To determine the impact of these nuclear inclusions on cell fate, we assessed cell proliferation by performing quantification of PCNA staining of HBsAg-Tg mice at 9 months of age, when the maximum vacuolated nuclei were observed and prior to the onset of hepatocellular carcinoma in this model. A subset of nuclei containing inclusions and normal nuclei were clearly PCNA positive (**Fig. 5A**). However, quantification revealed an increasing trend of PCNA positive vacuolated nuclei compare to normal nuclei, suggesting a correlation between the nuclear inclusions and cell proliferation (**Fig. 5B**). Almost none of these cells were TUNEL assay positive (**Fig. 5C**).

### Vacuolated nuclei in HBsAg-Tg liver are not associated with cell death or senescence

We wanted to know, whether non-proliferating (PCNA negative) hepatocytes with vacuolated nuclei were undergoing senescence or apoptosis. A previous study suggests that nuclear vacuolization is indicative of senescence [7]. While we observed positive senescence associated beta-galactosidase staining in frozen liver section, almost none of vacuolated nuclei coincided with positive staining (**Fig. 5D–F**.). This indicates that the cells containing vacuolated nuclei in the HBsAg-Tg mouse liver are not senescent. Similarly we noted that the senescence/DNA damage response marker gamma H2A.X was not observed in vacuolated nuclei (**data not shown**).

### Vacuolated nuclei contain 4-hydroxynonenal adducts and N-acetyl cysteine reduces accumulation of vacuolated nuclei in HBsAg-Tg mice

**Figure 6:**
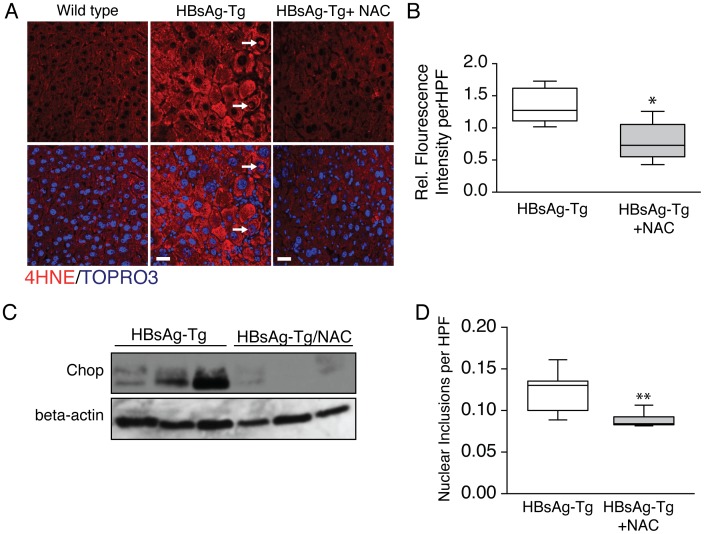
Oxidative stress may play a role in nuclear inclusion formation. (**A**) HBsAg-Tg mice liver show clear 4-HNE staining in hepatocytes including within nuclear inclusions of 9-month-old mice and 4-HNE levels are decreased with NAC treatment. (**B**) Quantification revealed a significant difference in 4-HNE levels in HBsAg-Tg mice treated with (n = 5) and without NAC (n = 5). (**C**) Decreased Chop protein levels in NAC-treated HBsAg-Tg mice (n = 3) versus HBsAg-Tg (n = 3) was confirmed by Western blot. (E) Nuclear inclusions were significantly reduced in NAC treated mice (n = 7) compare to HBsAg-Tg mice (n = 5). *,p<0.05; **,p<0.01, scale bar 20 µm.

An increased level of reactive oxygen species (ROS) leading to an imbalance of oxidation/reduction state, commonly referred to as oxidative stress, is a common feature of viral and non-viral hepatitis [25], [26], [27]. Increased levels of ROS species likely arises from the response to viral proteins and dysregulation of immuno-surveillance programs [28], [29]. It is well established that oxidative stress plays a key role in hepatocyte cell survival and proliferative responses during acute and chronic viral infection [30], [31]. Based on the proliferative phenotype we observed in hepatocytes with nuclear inclusions (**Fig. 5**), we wanted to explore the potential impact of oxidative stress on nuclear inclusions in our model of antigen-induced hepatitis. Oxidative stress in the liver, and other tissues, can result in the formation of 4-hydroxynoneal (4-HNE) adducts and over time can impact cell function. We observed increased levels of 4-HNE in the HBsAg-Tg mice at 9 months of age (**Fig. 6A–B**). Interestingly, we observed intense punctate immuno-histochemical staining in the nuclear inclusions of HBsAg-Tg (**Fig. 6A**). This prompted us to determine whether oxidative stress could play a role in nuclear inclusion formation. N-acetyl cysteine is an antioxidant used to reduce oxidative stress both experimentally and clinically during acute liver injury [32]. When 9–10 month old HBsAg-Tg mice were treated with NAC, we observed a marked reduction in 4-HNE adduct formation (**Fig. 6A–B**). NAC treatment also relieved endoplasmic reticulum (ER) stress as indicated by a decrease in the ER stress marker Chop (**Fig. 6C**). We observed a significant decrease in the number of nuclear inclusions in mice treated with N-acetyl-cysteine (**Fig. 6D**).

## Discussion

Our work demonstrates that a mouse model of hepatitis B surface antigen-induced injury recapitulates nuclear inclusions observed in clinical cases of viral hepatitis, fatty liver disease, and hepatocellular carcinoma. We observed that the inclusions were glycogen rich and contained cytoplasmic material. The inclusions increased in a time dependent manner and correlated with increased oxidative stress. PAS positive and calnexin-positive staining indicates that the inclusions are of cytoplasmic origin and contain components of the endoplasmic reticulum. PAS positive material (likely glycogen) in the inclusions is consistent with what has been observed in previous histological assessments [7]. We observed that the inclusions were consistently negative for the chromatin marker histone H3 and therefore rule out a chromatin or intranuclear origin for these inclusions. It is important to note, that we were not able to confirm if the HBsAg itself induces the formation of the nuclear inclusions.

Contrary to a recent analysis of nuclear inclusions in various human liver specimens, we did not observe activation of the senescence program in HBsAg-Tg hepatocytes containing nuclear inclusions (**Fig. 6D–F**) [7]. In contrast, we did observe a substantial fraction of hepatocytes with nuclear inclusions that were positive for the proliferation marker PCNA (**Fig. 6A**). Consistent with a previous report using transgenic mice over expressing hepatitis B antigens, these PCNA positive cells were atypically large and displayed a dysplastic phenotype (**Fig. 1** and [33]). We did not observe TUNEL staining in these cells suggesting that nuclear inclusions are not indicative of cell death (**Fig. 6B**). Differences could be attributed to the relative lack of senescence in the HBsAg-Tg model at the time points measured or a general resistance to the senescence program in these animals.

Large cell change/dysplasia (LCC/D) of hepatocytes in this mouse model may be indicative of pre-malignancy [34], [35]. LCC/D is defined as atypical hepatocytes, which are larger than normal/adjacent hepatocytes with large nucleus but normal nuclear to cytoplasmic ratio. These cells clearly exhibit nuclear atypia with marked pleomorphism, and prominent nucleoli. In our analysis, most hepatocytes with nuclear inclusions demonstrated characteristic LCC/D and where PCNA positive. This is indicative of a proliferative/regenerative phenotype for these cells and suggests that they are the likely precursors of HCC that develops in these animals at 12–18 months of age.

Hepatocytes may proliferate or undergo senescence in response to oxidative stress [36], [37]. We identified a strong correlation between hepatocyte nuclear inclusions and oxidative stress in HBsAg-Tg mice. 4-HNE adducts where highly expressed throughout the liver of HBsAg-Tg mice and were observed within the nuclear inclusions of these mice. NAC treatment effectively reduced oxidative stress levels and reduces 4-HNE adduct formation. This also resulted in a decrease in the number of nuclear inclusions in HBsAg-Tg mice. Our findings of increased oxidative stress and ER stress in the HBsAg-Tg strain are consistent with altered antioxidant and ER stress observed in other hepatitis b antigen transgenic mice [20], [38] [39].

While viral antigen production is not unique to the histopathological observation of nuclear inclusions, ER stress generated by antigen expression and accumulation could influence inclusion formation. ER stress is a common theme in chronic liver disease and is observed in the HBsAg-Tg mice. A Western blot for the ER stress marker Chop also indicated ER stress in HBsAg-Tg mice and this was reduced in NAC treated HBsAg-Tg mice compared to control transgenic mice. Further studies focused on ER stress and nuclear inclusions may provide new insights on the origins and impact of these structures.

In addition to the molecular pathways involved, an important concept to consider is the physical changes on cell shape associated with LCC/D. Increased volume of the nucleus may depend on DNA content (via increased ploidy) and/or changes in cytoskeletal structures in response to increased cytoplasmic volume [40]. Force generated by the expansion of the cell during LCC/D could lead to transient deformations of the nucleus resulting in the inclusions and pseudo-inclusions we report in HBsAg-Tg mice and that others have observed in human liver tissue. Maintaining integrity of the nuclear envelope (evidenced by a lack of diffusion of nuclear and cytoplasmic material) may likely be essential for hepatocytes with nuclear inclusions to remain viable. Furthermore, it remains unclear on how glycogen deposits accumulate in these inclusions. It will be important to develop systems to determine the composition, kinetics, and impact the inclusions have on hepatocyte fate both in vitro and in vivo.

Our results indicate that nuclear inclusions in LCC/D can be reproduced in a mouse model of hepatitis B antigen-induced injury. Furthermore, our data suggests that the formation of these inclusions is dependent on oxidative stress. The HBsAg-Tg provides a unique opportunity to explore the biological implications of these unique cellular structures. It remains unclear how these inclusions form, whether they are transient or prolonged formations, and the long-term consequences they have on cell fate.
